# Biomass-Derived Carbon Quantum Dots via Semi-Hydrothermal Processing: Linking Surface Chemistry, Colloidal Stability, and Photocatalytic Mineralization Performance

**DOI:** 10.3390/nano16120731

**Published:** 2026-06-12

**Authors:** Gamze Sak, Şeyda Taşar, Gülbeyi Dursun

**Affiliations:** Department of Chemical Engineering, Faculty of Engineering, Fırat University, 23119 Elazığ, Türkiye; gsak@firat.edu.tr (G.S.); gdursun@firat.edu.tr (G.D.)

**Keywords:** carbon quantum dots (CQDs), biomass valorization, semi-hydrothermal synthesis, photocatalytic degradation, total organic carbon (TOC) removal

## Abstract

In this study, carbon quantum dots (CQDs) were synthesized from various lignocellulosic and hemicellulosic biomass precursors via a semi-hydrothermal torrefaction process, and their structural, optical, colloidal, and photocatalytic properties were systematically investigated. Biomass sources including Oriental thuja cone (*Thuja orientalis*), sawdust, tea waste, apricot kernel shell, walnut shell, sugar beet pulp, hazelnut residue, soybean residue, and chitosan were used to evaluate the effect of precursor composition on CQDs characteristics. UV–Vis spectroscopy confirmed the formation of CQDs in all samples, exhibiting characteristic π–π* and n–π* transitions, while significant variations in absorption intensity and spectral behavior were observed depending on biomass type. Dynamic light scattering and zeta potential analyses revealed that most CQDs exhibited aggregation tendencies, with limited systems showing improved colloidal stability due to electrostatic and/or steric stabilization. The synthesized CQDs were combined with TiO_2_ and their influence on the photocatalytic degradation of Reactive Black 5 under UV irradiation was investigated. Although high decolorization efficiencies (85–98%) were achieved, total organic carbon removal remained lower (2.6–41.4%), indicating incomplete mineralization. The highest mineralization efficiencies were observed for TiO_2_ systems modified with sawdust- and thuja-derived CQDs. Overall, the results demonstrate that the photocatalytic performance of CQDs-modified TiO_2_ systems is governed not only by optical properties but also by surface functionalization, colloidal stability, and charge carrier dynamics. The findings highlight the critical role of biomass composition in determining CQD properties and provide a comparative framework for designing sustainable nanomaterials for environmental applications.

## 1. Introduction

Carbon nanomaterials have attained a significant position among next-generation functional materials in recent years due to their ability to tailor surface chemistry and morphology to desired properties, their high specific surface areas, and their advanced optical, electrical, and mechanical properties. These properties enable the widespread use of carbon nanomaterials across a wide range of disciplines, including advanced materials, energy technologies, environmental engineering, and bioengineering [1,2,3]. The broad application potential of carbon nanomaterials stems from the structural diversity resulting from the combination of sp^2^ and sp^3^ hybridized carbon atoms in various crystalline and amorphous arrangements [4].

Based on these structural differences, carbon nanomaterials are classified into forms such as zero-dimensional fullerenes, one-dimensional carbon nanotubes (CNTs), two-dimensional graphene, and three-dimensional diamond and graphite [3]. Within this classification, carbon quantum dots (CQDs), which fall under the category of zero-dimensional carbon nanomaterials, stand out as carbon-based nanostructures with semiconductor properties. CQDs were first discovered in 2004 during the purification of single-walled carbon nanotubes and have since become the subject of rapidly increasing research interest [1,2].

CQDs offer significant advantages for both scientific and industrial applications due to their superior photoluminescence properties, high chemical stability, low toxicity, biocompatibility, water solubility, and environmentally friendly nature [3,4,5]. These properties enable the potential use of CQDs in various fields such as bioimaging, catalysis, sensor technologies, healthcare applications, environmental monitoring, and renewable energy systems [6,7,8]. In photocatalytic applications, carbon quantum dots are commonly employed as co-catalysts or performance-enhancing components rather than as standalone photocatalysts. When coupled with semiconductor photocatalysts such as TiO_2_, CQDs can improve light harvesting, facilitate charge separation, suppress electron-hole recombination, and enhance the generation of reactive oxygen species. Therefore, the photocatalytic performance evaluated in this study reflects the behavior of CQDs-modified TiO_2_ systems rather than CQDs alone. Since the performance characteristics of CQDs are directly related to their size, surface functional groups, and structural arrangements, a systematic investigation of synthesis methods and the raw materials used is of great importance [9].

Generally, CQDs synthesis methods are classified into two main categories: “top-down” and “bottom-up” [10]. Top-down approaches rely on the fragmentation of large sp^2^ carbon structures into smaller-sized CQDs through physical or chemical processes. In this context, methods such as chemical oxidation, laser ablation [11], arc discharge [12], and electrochemical synthesis [13] are widely used. In contrast, bottom-up approaches aim to form CQDs through the controlled carbonization of small molecules or carbon-rich precursors; these include methods such as hydrothermal synthesis, microwave-assisted synthesis, and pyrolysis. These methods allow for more controlled adjustment of the size and morphology of CQDs [14].

However, a significant portion of traditional CQDs synthesis methods are not environmentally or economically sustainable due to the use of toxic solvents, hazardous chemicals, high energy requirements, and costly raw materials [15]. This situation has led researchers in recent years to adopt “green synthesis” approaches based on environmentally friendly, low-cost, and renewable raw materials. In this context, the hydrothermal synthesis method, which falls under the bottom-up category, stands out as an environmentally friendly approach based on the carbonization of organic carbon sources within Teflon-coated stainless steel autoclaves under closed, high-pressure conditions. Hydrothermal synthesis is widely preferred due to its ease of implementation, relatively low cost, and the ability to produce homogeneous CQDs with high quantum yield (QY) [16,17,18].

In hydrothermal synthesis, the type of carbon source used, in addition to reaction conditions, is one of the most critical parameters determining the structural and optical properties of the resulting CQDs. For this purpose, inorganic and organic carbon sources are commonly used. CQDs derived from inorganic carbon sources generally have low quantum yields, and additional processes such as heteroatom doping or surface passivation are required to enhance their performance [17,18,19,20]. In contrast, organic carbon sources are more widely preferred in CQDs synthesis due to their advantages of high yield, low cost, and sustainability. Among organic sources, animal waste, fruit and vegetable peels, and agricultural and industrial biomass waste play a significant role [21].

Biomass is one of the most abundant and renewable carbon sources in nature, with approximately 100 gigatons of biomass waste produced globally each year. The incineration of a large portion of this waste leads to serious environmental problems. In addition to biomass from the food industry, lignocellulosic waste derived from the wood and forestry industries also offers significant potential for the development of high-value-added nanomaterials [22,23]. In this context, biomass sources are considered a fundamental raw material for the sustainable synthesis of carbon quantum dots [24].

Lignocellulosic and hemicellulosic biomass are increasingly recognized as highly attractive carbon precursors for the synthesis of advanced carbon nanomaterials owing to their unique chemical composition and intrinsic carbon-rich structure. These renewable resources are primarily composed of cellulose, hemicellulose, and lignin, each contributing differently to carbon material formation. Cellulose and hemicellulose contain abundant oxygenated functionalities that promote dehydration, aromatization, and surface functionalization during thermal conversion, whereas lignin possesses a highly aromatic three-dimensional structure and elevated carbon content that facilitate the formation of graphitic carbon domains and stable carbon cores. Furthermore, lignin is considered the only naturally abundant renewable biopolymer intrinsically containing aromatic frameworks, making it particularly valuable for the production of functional carbon materials. The presence of hydroxyl, methoxy, carbonyl, and carboxyl groups in lignocellulosic biomass can also contribute to the generation of surface defects and functional groups that are beneficial for charge transfer, colloidal stability, and catalytic activity. In addition to their favorable chemical characteristics, lignocellulosic residues are inexpensive, widely available, environmentally sustainable, and generated in large quantities from agricultural, forestry, and food-processing industries. Recent studies have demonstrated that the intrinsic composition and molecular architecture of biomass precursors strongly influence the structural, optical, electronic, and catalytic properties of the resulting carbon nanomaterials, highlighting the importance of precursor selection in the design of biomass-derived carbon quantum dots [25,26,27,28].

Although there are numerous CQDs synthesis studies based on biomass in the literature, a large portion of these studies focus on the surface and morphological properties of CQDs derived from a single biomass source. However, studies that comparatively examine the structural and optical properties of CQDs synthesized from hemicellulosic and lignocellulosic biomass are limited in number.

The originality of this study stems from the systematic and comparative evaluation of lignocellulosic and non-lignocellulosic biomass sources with different chemical compositions and structural characteristics within a single synthesis framework. Unlike most previous studies that focus on the synthesis and characterization of CQDs from a single biomass source, the present work systematically compares nine biomass precursors with substantially different chemical compositions under identical processing conditions. By combining biomass characterization, CQDs surface chemistry, colloidal stability, optical behavior, and photocatalytic mineralization performance, this study establishes direct relationships between precursor composition and CQDs properties. The results provide a comparative framework for understanding how biomass chemistry governs the formation and environmental performance of biomass-derived carbon nanomaterials. Carbon quantum dots synthesized from Oriental thuja cone (*Thuja orientalis*), sawdust, tea waste, apricot kernel shell, walnut shell, sugar beet pulp, hazelnut residue, soybean residue, and chitosan were directly compared; thus clearly demonstrating the decisive influence of biomass origin on the structural and optical properties of CQDs as well as their colloidal stability and photocatalytic performance. Unlike studies in the literature that typically focus on a single type of biomass, this study offers a comprehensive approach encompassing different biomass classes, thereby filling a significant knowledge gap regarding the conversion of biomass and biomass waste into scalable, environmentally friendly, and high-value-added nanomaterials. Furthermore, the study provides a scientific framework directly aligned with the goals of green transformation, sustainable production, nanotechnology, and the circular economy outlined in the 12th Development Plan and the 2030 Industry and Technology Strategy.

## 2. Materials and Methods

In this study, carbon quantum dots were synthesized and characterized from various biomass wastes. The study was conducted in three steps. These are:

- Procurement and characterization of raw materials
- Production of CQDs via semi-hydrothermal pyrolysis
- Characterization of the produced CQDs.

**Raw Material Procurement:** The following were selected as biomass waste: Oriental thuja cone (*Thuja orientalis*), sawdust, tea waste, apricot kernel shell, walnut shell, sugar beet pulp, hazelnut residue, soybean residue, and chitosan. The waste biomass sources were obtained from local food processing plants, while chitosan was sourced from ADAGA A.Ş., (Antalya, Türkiye). a local company operating in the Antalya region. The collected biomass waste was first dried on polyethylene tarps under laboratory conditions.

**Characterization of Raw Materials:** The structural and morphological properties of the raw material sources were analyzed using Fourier-transform infrared spectroscopy (FTIR) and particle size distribution analyses.

**FTIR Analysis:** To identify the functional groups in the structure of the raw material sources, measurements were performed using a Shimadzu IRSpirit FTIR spectrometer (Shimadzu Corporation, Kyoto, Japan). Each measurement was conducted over a range of 400–4000 cm^−1^ with 45 scans. This method provided information about the chemical bonds and the structure of the compounds present in the raw material sources.

**Proximate Analysis:** This analysis includes ash content and volatile matter evaluation according to ASTM standards. Moisture content was determined using a Mettler LJ16 moisture analyzer (Mettler-Toledo AG, Greifensee, Switzerland) and correlated with the materials’ torrefaction properties.

**Elemental Analysis:** Elemental analysis for carbon (C), hydrogen (H), nitrogen (N), and sulfur (S) content was performed in accordance with ASTM standards using the LECO CHNS-932 elemental analyzer (LECO Corporation, St. Joseph, MI, USA). The analysis was conducted through a service contract with the Central Laboratory of Inönü University.

**Chemical Analysis:** The weight percentage compositions of the primary components constituting the chemical structure of the waste biomass samples (extractives, hemicellulose, lignin, and cellulose) were determined using analytical methods [29,30,31].

**Higher Heating Value (HHV):** The energy content of the raw material sources was calculated to assess their suitability for CQDs production via the semi-hydrothermal method. Empirical formulas were used for the calculation. The empirical formula used in this study was previously developed by the research group in another study [32]. 

**Figure 1:**
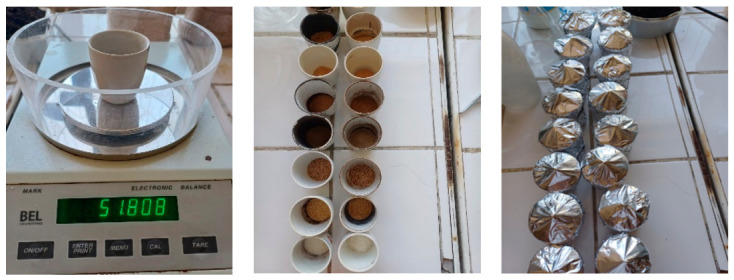
Weighing of raw materials and preparation for the semi-hydrothermal torrefaction process.

**Production of CQDs via semi-hydrothermal pyrolysis:** Raw materials (Oriental thuja cone (*Thuja orientalis*), sawdust, tea waste, apricot kernel shell, walnut shell, sugar beet pulp, hazelnut residue, soybean residue, and chitosan) were weighed at approximately 1 ± 0.02 g and placed into porcelain crucibles. The process was carried out after the initial weight (M_1_) of the empty crucibles, which had been pre-weighed to a constant weight, was recorded. The weight of the crucible and the sample was also measured using a precision balance and recorded as (M_2_). Distilled water with a solid-to-liquid ratio of 1/20 (g/mL) was added. The crucible lids were carefully closed. The crucibles were then wrapped in aluminum foil, ensuring no gaps remained—including at the lids—to prevent airflow or the escape of volatile components. After wrapping, prior to the semi-thermal torrefaction process and before placing them in the fixed-bed ash furnace, a hole was punched near the aluminum foil lid to allow for the escape of volatile components released during the thermal treatment. The crucibles prepared as described were placed in the ash furnace. The process was carried out under inert conditions in a nitrogen atmosphere, with the nitrogen flow rate set at 60 L/h (Figure 1).

**Figure 2:**
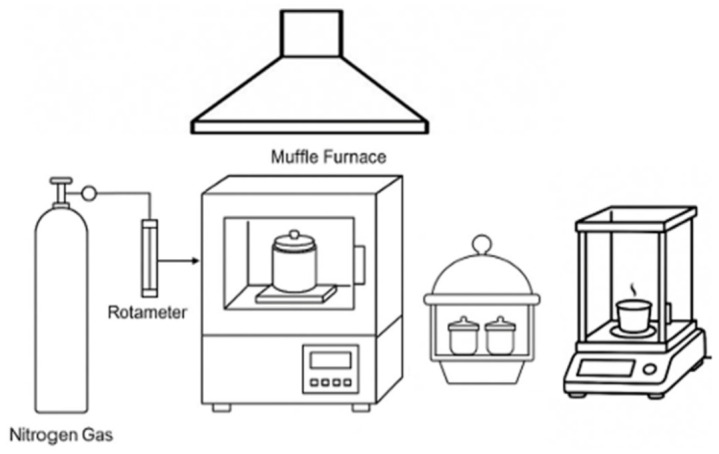
Schematic of the experimental setup.

The temperature of the ash furnace, operated under static conditions, was adjusted using the temperature control element located on the furnace’s temperature control panel. The schematic experimental setup is presented in Figure 1. The temperature programming device was set to reach 450 °C within 2 h and remain at the specified temperature for 1 h. The raw material sources were subjected to semi-hydrothermal processing at the specified temperature and duration. After the reaction was complete, the crucibles were removed from the ash furnace, placed in a desiccator, cooled to room temperature, and final weighings (M_3_) were performed. The semi-hydrothermal torrefaction yield (%) was calculated using the initial and final weights of the crucibles. This process was repeated twice while maintaining the solid-to-liquid ratio (Figure 2).

The samples obtained at the end of the process were transferred into centrifuge tubes with the addition of 40 mL of distilled water, and phase separation was performed using a Dragonlab brand centrifuge at 10,000 rpm for 10 min. The carbon quantum dots (CQDs) present in the supernatant were carefully separated and collected. The resulting supernatant was stored under laboratory conditions for TEM, photoluminescence imaging, particle size, and zeta potential analyses. The analyses performed on the CQDs and their conditions are presented below (Figure S1).

In this study, the term semi-hydrothermal refers to a water-assisted thermal conversion process conducted under oxygen-limited and partially confined conditions. Unlike conventional hydrothermal synthesis performed in sealed high-pressure autoclaves, the process operates without external pressure control. However, the presence of added water together with the confinement provided by the covered reaction vessel creates a localized moisture-rich environment that differs from conventional dry thermal treatment. Accordingly, the term semi-hydrothermal is used to distinguish the proposed process from both conventional hydrothermal synthesis and conventional dry torrefaction.

**FTIR Analysis:** To determine the structural changes in the CQDs samples following thermal treatment, measurements were performed using a Shimadzu IRSpirit FTIR spectrometer (Shimadzu Corporation, Kyoto, Japan). Each measurement was conducted over a range of 400–4000 cm^−1^ with a 45 scans.

**Zeta Potential:** The zeta potential analysis of CQDs samples was performed through a service contract with the Central Laboratory of Atatürk University (DAYTAM).

**Particle Size Distribution:** Particle size analysis was conducted in an aqueous medium to assess the hydrodynamic size distribution and colloidal behavior of CQDs. A PSS Nicomp Zeta Potential/Particle Sizer 380ZLS (Particle Sizing Systems, Santa Barbara, CA, USA) was used to measure the size distribution, providing critical information about the material’s particle structure and distribution homogeneity [33].

**Preparation of CQDs/TiO_2_ Photocatalytic Composites:** Prior to the photocatalytic degradation experiments, CQDs/TiO_2_ composites were prepared using a wet impregnation method. TiO_2_ (P25) was dispersed in 50 mL of an ethanol–water mixture (1:1, *v*/*v*) and stirred magnetically for 30 min to obtain a homogeneous suspension. Subsequently, a carbon quantum dot (CQD) solution corresponding to 5 wt.% of the TiO_2_ mass was added dropwise under continuous stirring. The resulting suspension was further stirred at room temperature for 2 h to facilitate the interaction between CQDs and the TiO_2_ surface. To improve the dispersion and promote a more uniform distribution of CQDs on TiO_2_ particles, the suspension was then subjected to ultrasonic treatment using a Bandelin ultrasonic homogenizer (20 kHz, 100 W) for 1 h. The resulting CQDs/TiO_2_ composites were used directly in the photocatalytic degradation experiments.

**Photocatalytic Degradation Test:** Carbon quantum dots derived from biomass were combined with TiO_2_, which served as the primary photocatalyst. The role of the CQDs was to modify and enhance the photocatalytic performance of the TiO_2_ system. Therefore, the photocatalytic degradation experiments evaluated the behavior of CQDs-modified TiO_2_ systems rather than CQDs alone. The degradation performance of these systems was investigated using a Reactive Black 5 dye solution prepared in a synthetic medium. The photocatalytic tests were conducted in a laboratory-scale 500 mL cylindrical glass reactor. The reactor system was mounted on a magnetic stirrer, and mixing was maintained by supplying air at a flow rate controlled by a flow meter. A cooling jacket connected to a circulating water bath was installed on the outer surface of the reactor to maintain a constant temperature. The system was equipped with a dual-mode light source capable of emitting light in both the visible and UV regions (UV-C, λ = 280 nm, 21 W) (Figure S2).

Prior to the reaction, the dye solution and photocatalyst mixture were left to pre-mix in the dark for 30 min to ensure adsorption–desorption and thermal equilibrium. Subsequently, the light source was activated to initiate degradation. The experimental parameters for the photocatalytic degradation are provided in the table below. During the experiments, samples were taken at specific time intervals, and the change in absorbance was measured using a UV-Vis spectrophotometer (λmax = 597 nm), and the degradation efficiency (%) was calculated. To analyze the degradation level of organic carbon, total organic carbon (TOC) analyses were conducted to monitor the degree of mineralization in the system [34,35,36].

For UV-Vis spectrophotometer measurements, samples were collected every 15 min for the first hour and every 30 min thereafter from the photodegradation reactor. Absorbance values were measured at a wavelength of λmax = 597 nm, and concentrations were calculated using a calibration curve. The removal efficiencies were calculated using the following formula based on the UV-Vis measurement values of the samples taken over time after the experiment. Here, C_0_: initial dye concentration (ppm), C: dye concentration after removal (ppm).

(1) $$\%Yield=\frac{C_{0}-C}{C_{0}}\times 100$$

Total organic carbon (TOC) is a technique used to measure the amount of organic matter in a water sample. In this study, the total organic carbon content of the post-degradation samples was measured using a Shimadzu TOC-L instrument (Shimadzu Corporation, Kyoto, Japan), and the mineralization value was calculated as the percentage removal efficiency (Table 1).

## 3. Results and Discussion

In this study, carbon quantum dots (CQDs) were synthesized using a semi-hydrothermal method with different lignocellulosic and hemicellulosic biomass sources, and the structural and photocatalytic properties of the obtained nanostructures were comparatively investigated. In this context, the proximate, chemical, and elemental analysis results of the biomass were evaluated; subsequently, the obtained CQDs were characterized via FTIR, particle size distribution, and photocatalytic degradation analyses. The results indicate that biomass composition plays a decisive role in CQDs formation and performance.

### 3.1. Characterization Results of Biomass Sources

**Figure 3:**
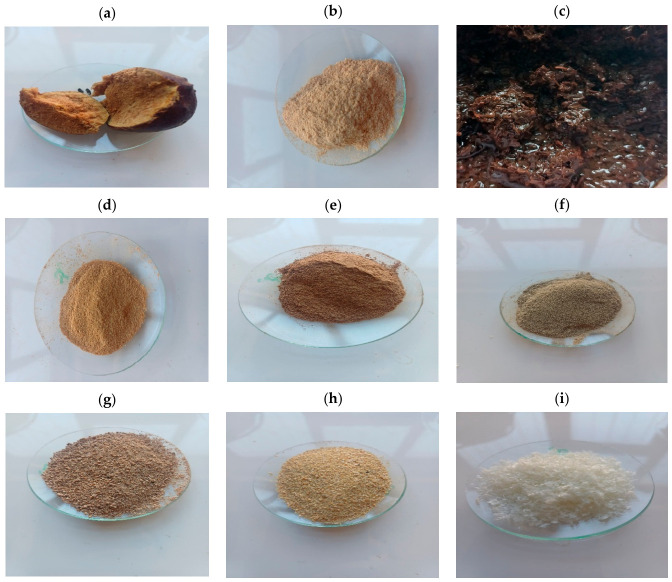
Biomass samples used in CQDs production: (**a**) Oriental thuja cone, (**b**) sawdust, (**c**) tea waste, (**d**) apricot kernel shell, (**e**) walnut shells, (**f**) sugar beet pulp, (**g**) hazelnut residue, (**h**) soybean residue, (**i**) chitosan.

Figure 3 presents the visual appearances of the biomass sources used in CQDs synthesis. The images reveal significant differences in the physical structure of the biomass samples. Woody biomass sources such as Oriental thuja cone and sawdust exhibit a fibrous lignocellulosic structure, whereas tea waste and beet pulp possess a looser and more porous morphology. In contrast, hard-shelled biomass sources such as walnut shells and apricot pits exhibit a more compact and lignin-rich structure. These structural differences can directly influence the carbonization behavior of the biomass and the morphology of the resulting carbon nanostructures.

After examining the visual characteristics of the biomass samples presented in Figure 3, the proximate, chemical, and elemental analysis results of the biomasses were considered together to evaluate their carbon quantum dot formation potential. The proximate analysis results provided in Table 2 reveal significant differences in the volatile matter, fixed carbon, and ash contents of the biomass samples. The volatile matter content was observed to range from 67.86% to 79.00%. The highest volatile matter content was determined in sawdust (79.00%), while this value was found to be 67.86% in beet pulp. A high volatile matter content can lead to the formation of more gas and volatile intermediate products during thermal conversion, thereby facilitating the formation of smaller carbon nuclei during the carbonization process. This finding is consistent with previous studies indicating that the separation of volatile components during the thermochemical conversion of lignocellulosic biomass can lead to the formation of smaller and more homogeneous carbon nanostructures [37].

In contrast, the fixed carbon content is higher in walnut shells (28.74%) and hazelnut residue (28.34%). A high fixed carbon ratio indicates that aromatic carbon structures are more dominant in the biomass structure, which may contribute to the development of the CQDs core structure by supporting the formation of sp^2^-hybridized carbon networks during carbonization. This finding is consistent with the literature reporting that lignin-rich biomasses form more stable aromatic structures during the carbonization process and strengthen the core structure of carbon nanomaterials [38].

Chemical analysis results (Table 3) indicate that the lignocellulosic composition of the biomass is consistent with the proximate analysis findings. It was determined that the biomass samples with the highest cellulose content were sawdust (43.2%) and Oriental thuja cone (42.5%). Polysaccharide structures such as cellulose and hemicellulose break down during thermal conversion to form oxygen-containing intermediates, which can contribute to the formation of functional groups such as hydroxyl, carbonyl, and carboxyl on the CQDs surface. This finding is consistent with studies reporting that the surface functionalization of biomass-derived CQDs largely originates from oxygen-containing compounds derived from cellulose and hemicellulose [38]. In contrast, biomass sources such as walnut shells (39.8%) and hazelnut residue (31.7%) are observed to have higher lignin content. Due to its aromatic structure, lignin forms more stable carbon structures during thermal processes, which may support the development of graphitic carbon cores. In this context, it is also emphasized in the literature that lignin-rich biomass promotes the formation of aromatic and sp^2^-hybridized carbon networks during the carbonization process, thereby strengthening the CQDs core structure [39]. Therefore, it is assessed that biomass with high lignin content may offer an advantage in terms of CQDs core formation.

Additionally, using proximate analysis data, the upper heating values calculated via the Parikh et al. (2005) [40] correlation were found to range from 19.17 to 21.11 MJ/kg. The highest HHV was calculated for walnut shells (21.11 MJ/kg), followed by hazelnut residue (20.97 MJ/kg) and chitosan (20.80 MJ/kg). The relatively high fixed carbon content of these biomass materials explains the higher calculated HHVs. Indeed, an increase in the fixed carbon ratio in biomass enhances energy density and is associated with higher calorific values [41] In contrast, HHVs were found to be relatively lower in beet pulp and soybean residue, which have high ash content. High ash content is known to be a significant parameter that reduces the energy content of biomass due to the increased presence of non-combustible inorganic components [42]. The results obtained are consistent with the typical calorific value ranges reported in the literature for lignocellulosic biomass, confirming the decisive role of biomass composition on energy content [43].

Elemental analysis results (Table 4) also provide important information regarding the carbonization potential of the biomass. It is observed that the carbon content varies in the range of 45.7–50.6%. The highest carbon content was determined in walnut shells (50.6%), which is consistent with the high lignin content. Similarly, the carbon content in hazelnut residue was determined to be 49.5%. In contrast, a significant increase in nitrogen content was observed in protein-rich biomass. For example, the nitrogen content was determined to be 7.9% in soybean residue and 6.5% in chitosan. It is known that biomass with high nitrogen content has the potential to form nitrogen-containing carbon structures during carbonization.

This finding is consistent with studies reporting that heteroatom doping in CQDs derived from nitrogen-rich precursors alters their surface chemistry and electronic properties [44]. It is expected that nitrogen-rich biomass sources, such as soybean residue and chitosan, will produce nitrogen-doped CQDs that significantly influence electronic properties and photocatalytic performance. Indeed, the literature reports that nitrogen doping improves the photoluminescence properties of CQDs, facilitates charge carrier separation, and supports electron transfer processes [45].

In general, when proximate, chemical, and elemental analysis results are evaluated together, it is observed that the compositional properties of the biomass are consistent with one another. The higher fixed carbon content in biomass with high lignin and carbon content (e.g., walnut shells and hazelnut residue) clearly demonstrates this consistency. Similarly, the higher volatile matter content in biomass rich in cellulose and hemicellulose is consistent with the thermal conversion behavior of the lignocellulosic structure. This finding is supported by studies showing that biomass composition plays a decisive role in product distribution and the development of carbon structure during thermochemical conversion processes [46]. These results indicate that biomass composition is a key parameter influencing both carbon core formation and the development of surface functional groups during carbon quantum dot synthesis. Indeed, the literature also highlights that the structural and surface properties of CQDs derived from different types of biomass vary significantly depending on their precursor composition [39].

**Figure 4:**
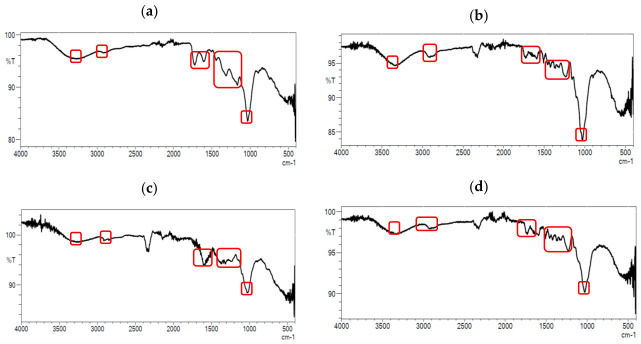

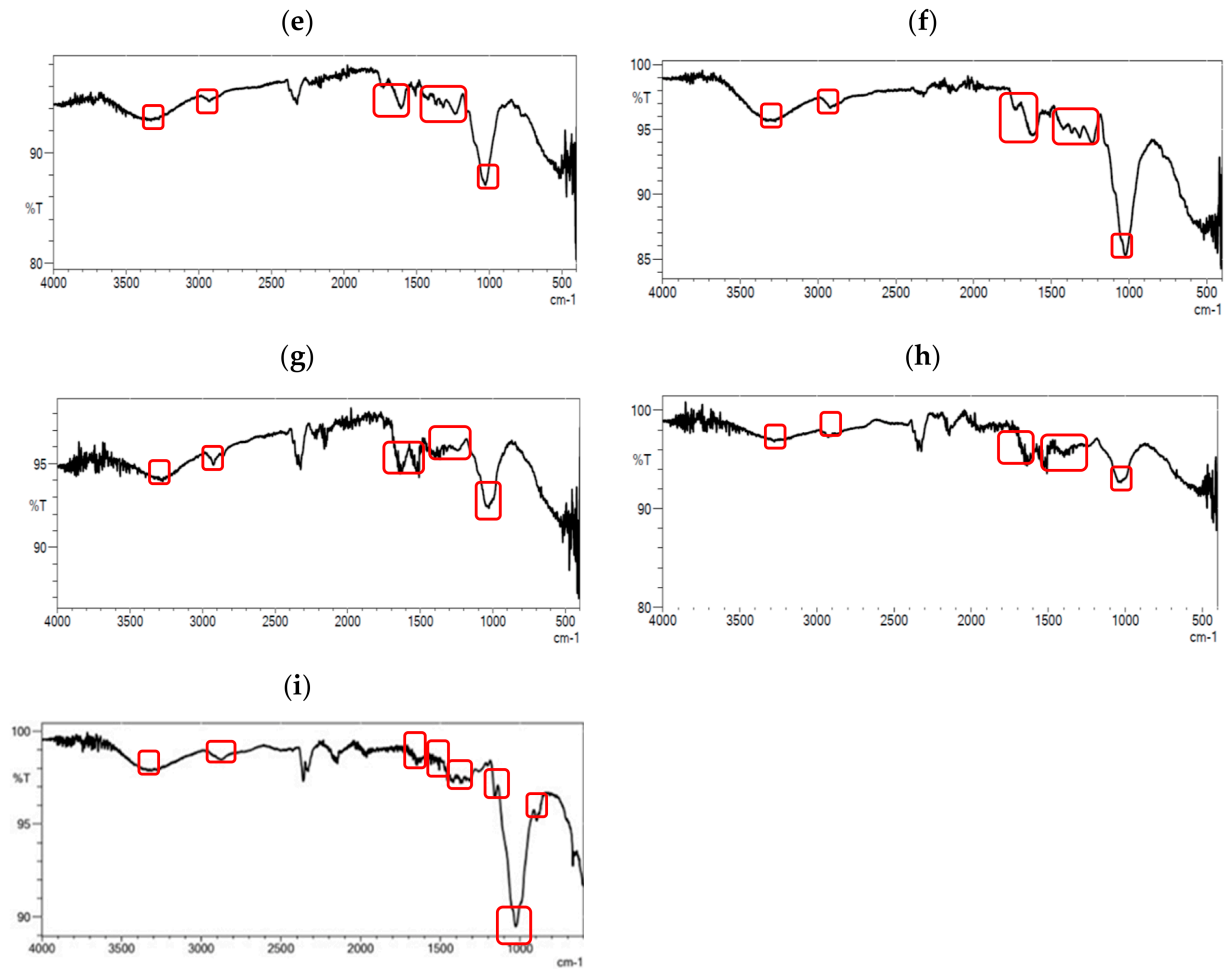
FTIR spectra of biomass samples: (**a**) Oriental thuja cone, (**b**) sawdust, (**c**) tea waste, (**d**) apricot kernel shell, (**e**) walnut shells, (**f**) sugar beet pulp, (**g**) hazelnut residue, (**h**) soybean residue, (**i**) chitosan.

Upon examination of the FTIR spectra of waste biomass samples, characteristic absorption bands specific to lignocellulosic biomass are observed in all samples (Figure 4). In particular, the broad band observed in the 3200–3600 cm^−1^ range corresponds to the stretching vibrations of hydroxyl (–OH) groups; this band originates from the hydroxyl groups present in the cellulose, hemicellulose, and lignin components of the biomass structure. This band is clearly observed in all biomass samples. The bands observed around 2920–2850 cm^−1^ in the spectra are associated with aliphatic C–H stretching vibrations and represent methyl and methylene groups in the biomass structure. These bands are particularly more pronounced in woody biomass (Oriental thuja cone and sawdust). The absorption bands observed in the 1700–1600 cm^−1^ range are associated with the vibrations of carbonyl (C=O) and aromatic C=C bonds. These bands indicate the presence of aromatic rings in the lignin structure. These bands are particularly pronounced in biomass with high lignin content (such as walnut shells and hazelnut residues). The bands in the 1500–1200 cm^−1^ range are associated with aromatic skeleton vibrations and C–H bending vibrations. These bands are characteristic structural bands of lignocellulosic biomass. Additionally, the strong absorption bands observed around 1100–1000 cm^−1^ correspond to C–O and C–O–C stretching vibrations, indicating the presence of glycosidic bonds in cellulose and hemicellulose structures. A weak absorption feature observed in the 2300–2400 cm^−1^ region is attributed to atmospheric CO_2_ interference during FTIR measurement and is not considered a characteristic functional group. These FTIR bands are consistent with the characteristic vibration regions widely reported in the literature for different types of biomass (wood waste, agricultural residues, and bark structures) [47,48,49,50].

In general, these bands observed in all biomass samples confirm the presence of cellulose, hemicellulose, and lignin, which are the fundamental components of the lignocellulosic structure. The obtained FTIR results are consistent with the chemical composition analyses of the biomass and indicate that the raw materials used contain functional groups suitable for carbonization and carbon nanostructure formation. This finding is also consistent with studies reporting that the surface functional groups of biomass-derived CQDs largely derive from the chemical structure of the precursor biomass.

Upon examination of the FTIR spectrum of the chitosan sample presented in Figure 4i, the broad absorption band observed in the 3000–3700 cm^−1^ region is attributed to the overlapping stretching vibrations of hydroxyl (–OH) and amino (–NH_2_) groups present in the chitosan structure. This broad band reflects the hydrophilic nature of chitosan and indicates the extensive intermolecular and intramolecular hydrogen bonding within the polymer network. Weak absorption features observed in the 2400–2300 cm^−1^ region are most likely associated with atmospheric CO_2_ interference or background correction effects and are therefore not considered characteristic bands of chitosan.

The weak bands observed around 2920–2870 cm^−1^ are assigned to the asymmetric and symmetric stretching vibrations of aliphatic C–H bonds (–CH_2_ and –CH_3_ groups), respectively. The absorption peak located near 1640 cm^−1^ corresponds to the amide I band and is associated with C=O stretching vibrations originating from residual N-acetyl groups remaining in the partially deacetylated chitosan structure. In addition, the band observed around 1550 cm^−1^ is attributed to the amide II band, arising mainly from N–H bending vibrations coupled with C–N stretching vibrations, confirming the presence of amino functionalities within the chitosan backbone.

The band observed around 1320 cm^−1^ is associated with amide III vibrations and C–N stretching of amino groups. A characteristic absorption peak appearing near 1150 cm^−1^ is assigned to the asymmetric stretching vibration of the C–O–C bridge in the polysaccharide structure. Furthermore, the strong absorption bands observed in the 1075–1020 cm^−1^ region are attributed to C–O stretching vibrations of the glucosamine units and glycosidic linkages constituting the chitosan backbone. These bands are considered characteristic fingerprints of the polysaccharide structure of chitosan.

Overall, the FTIR spectrum confirms the characteristic polysaccharide structure of chitosan containing abundant hydroxyl and amino functional groups. The presence of these functional groups is responsible for the hydrophilic character, adsorption capability, and chemical reactivity of chitosan, making it a promising material for environmental, catalytic, and biomedical applications [51,52].

### 3.2. Thermal Processing Efficiency and CQDs Characterization

An analysis of the thermal processing yields presented in the graph reveals that the yield values of the biomass samples ranged from 50.40% to 67.29%. The highest yield was obtained from sawdust (67.29% ± 0.95), followed by beet pulp (62.85% ± 0.82) and Oriental thuja cone (62.63% ± 0.80). This finding can be attributed to the relatively high fixed carbon contents and the thermal stability of the lignocellulosic structures of these biomass materials, as determined by proximate analysis results [53].

Medium-level yield values were determined for apricot kernel shell (61.91% ± 0.75) and hazelnut residue (60.00% ± 0.78). The higher hemicellulose fraction in these biomass materials contributes to the formation of volatile compounds by breaking down more easily at torrefaction temperatures, thereby increasing mass loss [54].

The lowest yields were observed in soybean residue (50.40% ± 0.60) and walnut shells (53.89% ± 0.65). In particular, the high nitrogen content and protein structures identified in the elemental analysis of soybean residue may lead to the formation of more volatile compounds during thermal degradation, thereby causing a decrease in yield [55]. Overall, the results indicate that thermal processing yield is consistent with the volatile matter–fixed carbon distribution and chemical composition of the biomass as determined by proximate analysis. It is observed that yields are relatively high in woody biomass with a more stable lignocellulosic structure, whereas yields are lower in biomass with high protein and hemicellulose content due to thermal degradation [56] (Table 5).

### 3.3. Characterization Results of CQDs Samples Obtained from Different Biomass Sources

**Figure 5:**
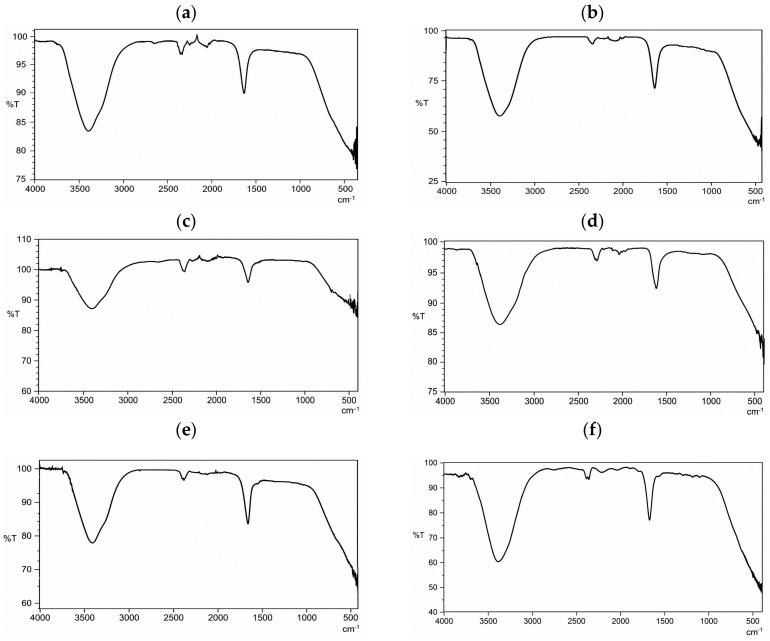

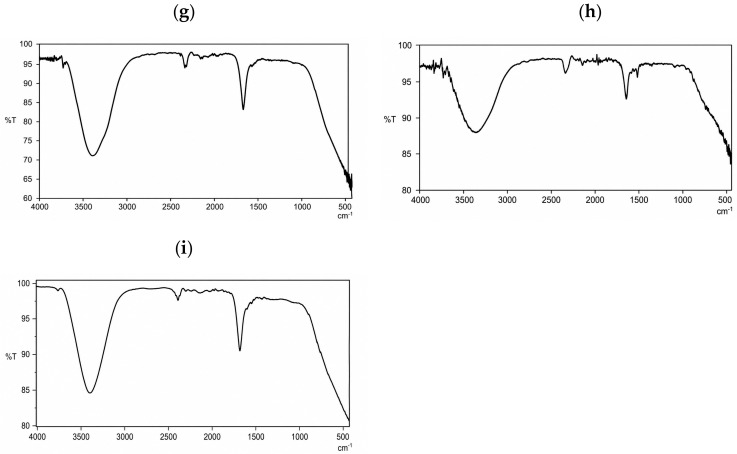
FTIR spectra of CQDs: (**a**) Oriental thuja cone, (**b**) sawdust, (**c**) tea waste, (**d**) apricot kernel shell, (**e**) walnut shells, (**f**) sugar beet pulp, (**g**) hazelnut residue, (**h**) soybean residue, (**i**) chitosan.

FTIR spectra of carbon quantum dots (CQDs) synthesized from different biomass sources are presented in Figure 5. Upon examination of the spectra, similar functional group bands are observed in all samples, indicating that CQDs derived from different biomass sources possess comparable surface chemistry. This finding is consistent with studies reporting that hydrothermal and semi-hydrothermal processes result in similar surface functionalization of CQDs despite differences in biomass feedstocks [38,39].

The broad absorption band observed in the 3200–3500 cm^−1^ region is attributed to the stretching vibrations of hydroxyl (O–H) and/or amine (N–H) groups. This band indicates the presence of oxygen-containing surface functionalities formed during the semi-hydrothermal conversion of biomass. These functional groups contribute to the hydrophilic character and good dispersibility of CQDs in aqueous media. A weak absorption feature observed around 2300–2400 cm^−1^ is attributed to atmospheric CO_2_ interference during FTIR measurement and was therefore not considered in the structural interpretation of the synthesized CQDs.

The weak bands observed around 2920–2850 cm^−1^ are associated with aliphatic C–H stretching vibrations, indicating the presence of residual aliphatic carbon structures originating from biomass components or formed during carbonization [39]. The absorption band observed in the 1700–1600 cm^−1^ region is attributed to carbonyl (C=O) stretching vibrations and aromatic C=C skeletal vibrations. The presence of these bands suggests the formation of partially aromatized carbon structures and oxygen-containing surface groups during the semi-hydrothermal carbonization process.

Similar bands have been widely reported for biomass-derived CQDs and are considered characteristic features of carbonized carbonaceous materials [43,57].

The bands observed in the 1450–1200 cm^−1^ region are associated with C–H bending vibrations as well as C–N and C–O bond vibrations. Furthermore, the strong absorption bands observed around 1100–1000 cm^−1^ are assigned to C–O and C–O–C stretching vibrations. These bands indicate the presence of oxygen-containing functional groups such as hydroxyl, ether, and epoxy groups on the CQDs surface. Such functionalities are known to influence the surface properties, chemical reactivity, and dispersion behavior of carbon quantum dots [39,45].

Overall, the FTIR spectra indicate that biomass is transformed into carbonaceous nanostructures containing abundant oxygen-containing surface functional groups during the semi-hydrothermal process. The presence of these functionalities supports the hydrophilic nature and surface activity of the synthesized CQDs. These observations are consistent with previous studies reporting that the surface chemistry of biomass-derived CQDs is dominated by hydroxyl, carbonyl, carboxyl, and ether-related functional groups [43,57].

Additionally, when compared with the FTIR spectra of the initial biomass samples, the characteristic bands associated with polysaccharide and lignocellulosic structures become less pronounced, while oxygen-containing surface functionalities become more evident. This observation suggests that biomass undergoes carbonization and aromatization reactions during the semi-hydrothermal treatment, resulting in the formation of carbon quantum dots. Similar transformation mechanisms have been extensively reported in the literature for biomass-derived CQDs [37,43].

The particle size distribution of carbon quantum dots (CQDs) synthesized from various biomass precursors was systematically evaluated using dynamic light scattering (DLS), and the results are presented in Table 6. The results revealed significant differences in distribution behavior, aggregation tendency, and colloidal stability depending on the biomass source.

The Z-average values of all samples ranged from 897 nm to 3623 nm, which are significantly higher than the expected size range for CQDs (typically <10 nm). This discrepancy indicates that the measured values correspond to the hydrodynamic diameter of aggregated structures rather than the primary particle size. Such behavior is commonly observed in hydrothermally synthesized carbon-based nanomaterials, where insufficient surface stabilization leads to particle clustering in an aqueous environment.

Polydispersity index (PdI) values also supported these observations. Most samples exhibited high PdI values (0.65–1.00), confirming broad and heterogeneous size distributions. In particular, CQDs derived from tea waste exhibited an extreme PdI value of 1.00, indicating significant instability and highly polydisperse behavior.

Similarly, samples obtained from juniper wood and walnut shells exhibited PdI values above 0.85, indicating significant aggregation and weak colloidal stability.

In contrast, CQDs derived from hazelnut shells (PdI ≈ 0.68) and soybean residue (PdI ≈ 0.65) exhibited relatively lower PdI values, demonstrating more homogeneous size distributions and improved dispersion properties. The chitin-based system also exhibited moderate polydispersity (PdI ≈ 0.70) with low standard deviation values, indicating reproducible and relatively stable particle formation.

Analysis of density-based size distributions revealed three distinct distribution models:

Monomodal but aggregation-dominated systems (walnut shell, sugar beet pulp), where a single peak (~500–650 nm) accounted for nearly 100% of the distribution. Despite the apparent uniformity, large Z-average values indicate that these systems consist primarily of aggregate clusters, masking the true nanoscale nature of the CQDs. Systems with multimodal, complex size distributions (tea residue, sawdust) are characterized by the presence of multiple peaks, including large aggregates exceeding 5 µm.

These systems exhibit unstable distributions due to the simultaneous presence of primary particles and large agglomerates. Relatively narrower and more stable distributions (walnut shell, soybean residue) feature a dominant peak (~400–600 nm) alongside a small fraction of smaller particles (~20–50 nm), suggesting that nano-scale structures are partially preserved.

The observed differences can be attributed to the intrinsic chemical composition of the biomass precursors. Biomass with higher protein, lipid, and oxygen-containing functional groups (e.g., soybean residue, and walnut shell) likely reduces aggregation by providing better surface passivation and electrostatic stabilization. In contrast, lignin-rich and highly aromatic precursors (e.g., tea waste, woody biomass) tend to produce more hydrophobic carbon structures with limited surface functionality; this leads to stronger interparticle interactions and aggregation. Additionally, the hydrothermal torrefaction process can cause varying degrees of carbonization and surface oxidation, which directly affect the zeta potential and colloidal stability. The absence of sufficient surface functional groups or stabilizing agents may have caused secondary aggregation, as evidenced by the large hydrodynamic diameters observed in most samples.

Overall, the results indicate that biomass selection plays a critical role in determining the distribution quality and stability of CQDs. Among the tested samples, CQDs derived from walnuts and soybeans exhibited the most suitable distribution behavior, while tea waste and lignocellulosic biomass showed the highest tendency toward aggregation.

It should be emphasized that DLS provides hydrodynamic size measurements and is highly sensitive to aggregation. Therefore, the true primary particle size of CQDs is expected to be significantly smaller. To accurately determine the intrinsic size and morphology of nanoparticles, complementary techniques such as transmission electron microscopy (TEM) are required. This finding is consistent with studies reporting that DLS measurements reflect the hydrodynamic diameter of aggregated structures and that the actual nanoparticle size is generally smaller [39,45].

**Figure 6:**
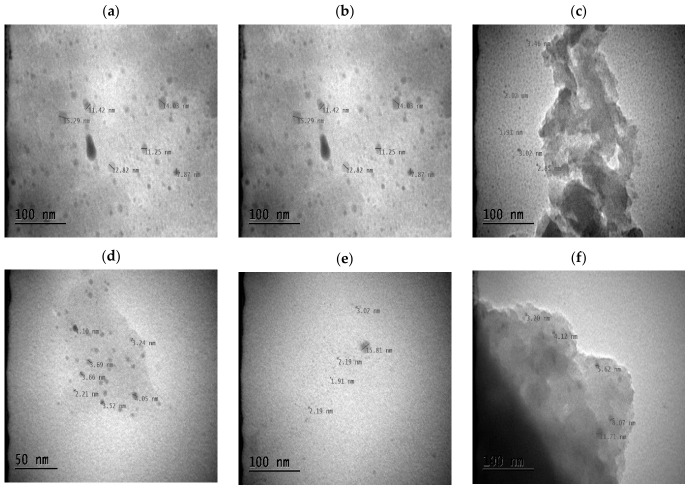

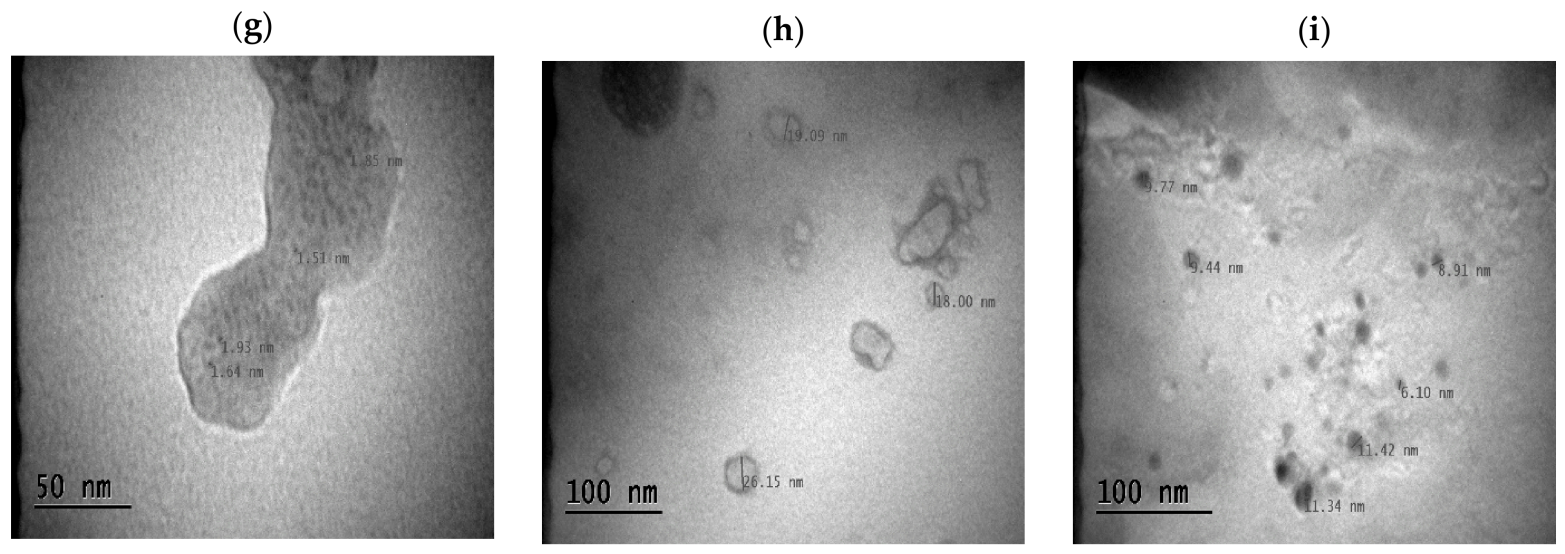
Representative TEM images showing the morphology and particle size distribution of carbon quantum dots (CQDs) synthesized via the semi-hydrothermal conversion of different biomass precursors: (**a**) Oriental thuja cone, (**b**) sawdust, (**c**) tea waste, (**d**) apricot kernel shell, (**e**) walnut shell, (**f**) sugar beet pulp, (**g**) hazelnut residue, (**h**) soybean residue, and (**i**) chitosan.

The morphological characteristics of the synthesized carbon quantum dots (CQDs) were investigated by transmission electron microscopy (TEM), and the representative TEM images are presented in Figure 6.

Examination of the micrographs revealed that the CQDs predominantly exhibited spherical or quasi-spherical morphology with a relatively uniform nanoscale distribution. Based on direct measurements from the TEM images, the particle sizes were found to range approximately between 2 and 20 nm.

The observed particle size range is consistent with the dimensions commonly reported for carbon quantum dots in the literature and confirms the successful synthesis of nanoscale carbon nanostructures from biomass-derived precursors. Furthermore, the nanosized dimensions observed via TEM provide direct evidence supporting the classification of the synthesized materials as carbon quantum dots.

Although the CQDs were generally well dispersed, localized agglomerated regions were also observed in some areas of the TEM images. These aggregations may be attributed to intermolecular interactions among surface functional groups, particularly hydroxyl, carboxyl, and other oxygen-containing moieties present on the CQDs surface. Hydrogen bonding and van der Waals interactions between neighboring particles are known to promote partial aggregation during drying and sample preparation for TEM analysis.

The particle sizes determined by TEM were considerably smaller than the values obtained from dynamic light scattering (DLS) measurements. This discrepancy is expected because DLS measures the hydrodynamic diameter of particles suspended in solution rather than the physical diameter of individual nanoparticles. Consequently, DLS measurements include the contribution of surface functional groups, solvation layers, and possible aggregated structures present in the suspension. Therefore, the DLS results primarily reflect the colloidal stability and aggregation behavior of the CQDs, whereas TEM provides direct information regarding the dimensions of individual carbon dots. Similar differences between DLS-derived hydrodynamic diameters and TEM particle sizes have frequently been reported for biomass-derived CQDs in the literature.

Overall, the TEM results, together with the UV–Vis spectra showing characteristic π–π* and n–π* transitions, the photoluminescence behavior observed under UV irradiation, FTIR analyses, particle size distribution, and zeta potential measurements, collectively confirm the successful formation of carbon quantum dots through the semi-hydrothermal conversion of biomass-derived carbon precursors.

The surface charge and colloidal stability of carbon quantum dots (CQDs) derived from different biomass sources were evaluated via zeta potential (ZP) measurements, and the results are presented in Table 7. The results indicate that the zeta potential values of the samples vary over a wide range and that stability behavior depends not only on the magnitude of the surface charge but also on environmental conditions and surface chemistry. This finding is consistent with the literature highlighting that CQDs stability is a multi-parameter system [38,45].

In general, the zeta potential values of CQDs samples range from −35 mV to +6 mV. When evaluated according to stability criteria widely accepted in the literature, it is known that |ZP| ≥ 30 mV values indicate high electrostatic stability, while |ZP| < 20 mV values indicate poor stability. In this context, it was determined that the vast majority of the samples examined have low absolute zeta potential values and thus form electrostatically unstable systems [45].

In particular, for CQDs based on tea waste (Sample 3), walnut shells (Sample 5), and sugar beet pulp (Sample 6), zeta potential values were observed to be below −10 mV, indicating that electrostatic repulsion between particles was insufficient and that the tendency toward aggregation was high. Additionally, high conductivity values in these samples (particularly in Samples 5 and 6) caused the electrical double layer to collapse, further weakening the zeta potential effect and promoting aggregation. This behavior is consistent with studies reporting that an ionic environment reduces colloidal stability by decreasing the thickness of the electric double layer [38].

It was determined that the zeta potential values in CQDs based on Oriental thuja cone (Sample 1), sawdust (Sample 2), and chitosan (Sample 9) were in the range of approximately −12 to −15 mV, indicating that these systems possess weak electrostatic stability and exhibit a tendency toward aggregation. These results are consistent with the high hydrodynamic diameters and broad size distributions observed in these samples.

In contrast to the other samples, apricot kernel shell-based CQDs (Sample 4) exhibited positive zeta potential values (+4–6 mV). However, due to the low absolute values, the electrostatic stability was still considered limited. Nevertheless, the relatively lower aggregation observed in this sample indicates that stability cannot be explained solely by electrostatic interactions and that steric stabilization mechanisms may also play a role.

This finding is consistent with the literature reporting that surface functional groups and organic coating layers can provide stability through steric hindrance [39]. CQDs based on walnut hull (Sample 7), despite having a zeta potential of approximately −8 mV, exhibited relatively better dispersion behavior in DLS analyses. This suggests that system stability cannot be explained solely by zeta potential and that protective (capping) layers formed on the surface by biomass-derived organic components provide steric stabilization.

The most striking result was obtained for soybean residue-based CQDs (Sample 8). In this sample, zeta potential values were determined to be in the range of −32 to −35 mV, indicating strong electrostatic stability. The fact that the system remains stable despite high conductivity values indicates that the magnitude of the surface charge suppresses the effect of the ionic environment and that electrostatic repulsion effectively prevents aggregation. This finding is consistent with studies reporting that high absolute zeta potential values are decisive in preventing aggregation in colloidal systems [45].

Overall, the stability of CQDs systems can be explained by three distinct mechanisms: (i) electrostatic stabilization, (ii) steric stabilization, and (iii) weak or insufficient stabilization. These findings indicate that the chemical composition of the biomass source (particularly proteins, lignin, and surface functional groups) plays a critical role in determining the surface charge of CQDs and, consequently, their colloidal stability. Furthermore, it was concluded that electrostatic stability may decrease under high conductivity conditions, but sufficiently high zeta potential values can counteract this effect. This finding is consistent with studies highlighting the decisive influence of biomass composition on CQDs surface chemistry and stability [38,39].

The optical properties of carbon quantum dots (CQDs) synthesized via the hydrothermal torrefaction method from different biomass sources were investigated using UV–Vis absorption spectroscopy, and the results are summarized in Table 8. The observation of characteristic absorption bands in the UV region in all samples clearly indicates that CQDs formation was successfully achieved. This finding is consistent with studies reporting that biomass-based CQDs exhibit characteristic absorption in the UV region [39,45].

The absorption bands observed in the 250–260 nm range in all samples have been attributed to π–π* electronic transitions arising from C=C bonds in aromatic sp^2^ hybridized carbon structures. These bands confirm the presence of a graphitic carbon core in the CQDs and were observed more distinctly in the samples derived from juniper Oriental thuja cone, tea waste, chitosan, and hazelnut residue.

In contrast, the fact that these bands are wider and of lower intensity in the sawdust and apricot kernel shell samples indicates that a more amorphous carbon structure is dominant. These findings are consistent with the literature reporting that the optical properties of CQDs depend on the degree of carbonization and the regularity of the core structure [39,57].

In addition, a second absorption band or shoulder was observed in the 270–280 nm range in many samples. This band is associated with n–π* transitions arising from oxygen- and/or nitrogen-containing functional groups (C=O, –COOH, –OH, and –NH_2_) present on the surface. In particular, it was determined that this band is quite pronounced in walnut shell, soybean residue, and chitosan-based CQDs, and that surface functionalization has increased significantly.

This finding is consistent with studies reporting that functional groups containing heteroatoms on the CQD surfaces determine the electronic transitions and optical behavior [38,45].

Unlike all other samples, soybean and hazelnut residue-based CQDs exhibited spectra in which both π–π* and n–π* transitions were observed at high intensity, and absorption extended strongly into the visible region beyond 280 nm. This phenomenon is attributed to the presence of high surface defect density, rich functional group content, and advanced surface states. It has been observed that surface chemistry in such systems determines not only the optical properties but also the colloidal stability. This finding is consistent with studies reporting that surface conditions in CQDs control both optical properties and dispersion behavior [39,57].

In walnut shell-based CQDs, it was determined that π–π* transitions are weak, whereas n–π* transitions are dominant, indicating that surface functionalization is more dominant compared to the core structure. In the apricot kernel shell and sawdust samples, the relatively weak nature of both transitions has been attributed to a lower degree of carbonization and a more heterogeneous structure.

In all samples, it was observed that absorption gradually decreases beyond 280 nm and extends into the visible region. This behavior is the absorption tailing commonly reported in CQDs and is associated with surface defects, energy traps, and a heterogeneous electronic structure. It is also emphasized in the literature that such optical behaviors are directly related to the surface defects and energy level distribution of CQDs [38,57].

The negative absorbance values observed at certain wavelengths in the spectra are not physically meaningful and are related to baseline errors or instrument noise occurring during measurement. Therefore, the evaluation was conducted by considering the general trend of the spectra and the characteristic absorption regions.

Overall, the UV–Vis results clearly demonstrate that in all synthesized CQDs samples: a graphitic carbon core has formed, surface functionalization has occurred, and typical CQDs optical behavior is exhibited.

When evaluated in conjunction with DLS and zeta potential analyses, these findings indicate that the optical properties of CQDs are largely controlled by surface chemistry and that the biomass source plays a decisive role in these properties. This is consistent with studies highlighting the critical influence of biomass composition on the surface states and optical performance of CQDs [39,43].

**Figure 7:**
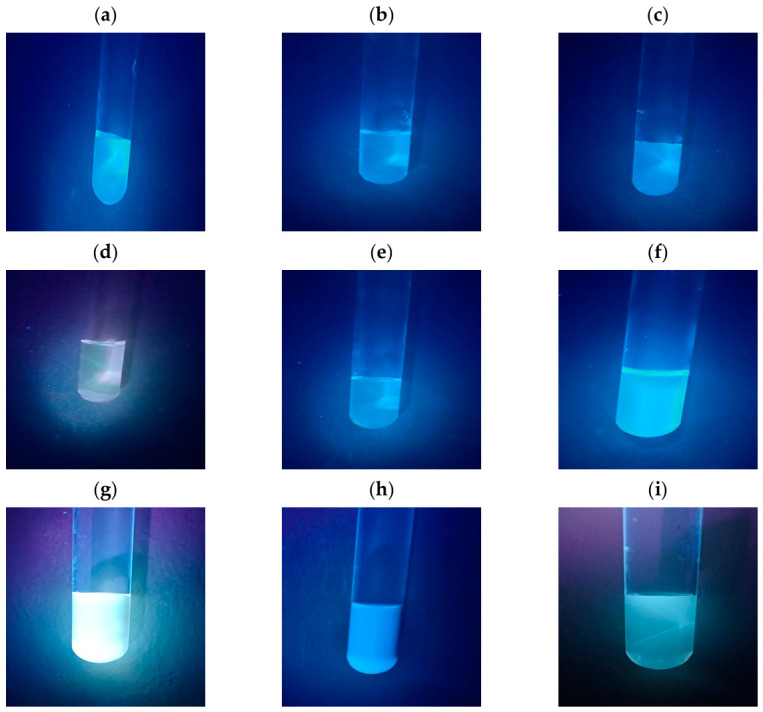
Comparative images of the photoluminescence behavior of carbon quantum dots (CQDs) synthesized via the semi-hydrothermal method from different biomass sources under UV light (λ = 365 nm): (**a**) Oriental thuja cone, (**b**) sawdust, (**c**) tea waste, (**d**) apricot kernel shell, (**e**) walnut shells, (**f**) sugar beet pulp, (**g**) hazelnut residue, (**h**) soybean residue, (**i**) chitosan. The figures demonstrate that the emission color, intensity, and colloidal distribution properties vary significantly depending on the type of biomass.

The photoluminescence images obtained under UV light, presented in Figure 7, reveal that the optical properties of CQDs synthesized from different biomass sources vary significantly. The observation of characteristic emission in the blue–turquoise region in all samples confirms the successful formation of CQDs. This finding is consistent with the literature reporting that biomass-based CQDs generally exhibit blue-region emission [38,39]. However, differences in emission intensity and color tone indicate that surface functionalization, particle size, and the level of heteroatom incorporation vary depending on the biomass origin. This finding is consistent with studies highlighting that the optical properties of CQDs are controlled by their surface states and chemical composition [45,57].

More homogeneous and intense blue emission was observed in CQDs derived from woody lignocellulosic biomass (particularly sawdust and Oriental thuja cone), a phenomenon that can be attributed to good surface passivation and a narrower size distribution. The fact that these samples also exhibit higher TOC removal efficiency suggests an indirect relationship between photoluminescence behavior and photocatalytic mineralization performance. This relationship is supported by studies reporting that surface defects and the degree of passivation in CQDs affect charge carrier separation and the formation of reactive species [43,57].

In contrast, it has been observed that the emission spectrum of CQDs derived from high-nitrogen-content biomasses (e.g., soybean residue and chitosan) shifts toward the green region. This phenomenon can be explained by the narrowing of the bandgap and changes in surface states due to nitrogen doping. The literature reports that nitrogen doping creates new energy levels in CQDs, shifting the emission wavelength to longer wavelengths [38,45].

The turbidity and opaque appearance observed in some samples indicate low colloidal stability and the occurrence of particle aggregation. While the increased light scattering effect in such systems may appear to enhance visible emission intensity, this situation poses a disadvantage in terms of photocatalytic performance. This behavior is consistent with studies reporting that aggregation can both mislead optical measurements and reduce catalytic performance by decreasing the active surface area [39].

Indeed, the fact that some samples with high color removal efficiency exhibit low TOC removal demonstrates that photoluminescence intensity alone is not a reliable indicator of photocatalytic mineralization performance. This finding is consistent with the literature indicating that there may not be a direct correlation between the optical properties of CQDs and their catalytic activity, and that surface reaction mechanisms are the determining factors [43,57].

Overall, the visual photoluminescence results obtained under UV light demonstrate that the optical properties of CQDs are directly related to biomass composition, surface chemistry, and colloidal stability, and support the decisive role of these parameters in photocatalytic performance. These findings clearly highlight that in CQDs design, not only optical properties but also surface functionalization and dispersion behavior must be taken into account [39,45].

It should be noted that the photocatalytic experiments were performed using CQDs-modified TiO_2_ systems rather than CQDs alone. In these systems, TiO_2_ served as the primary photocatalyst, while the biomass-derived CQDs acted as performance-enhancing components by influencing light absorption characteristics, charge separation efficiency, and reactive species generation. Therefore, the photocatalytic results discussed below reflect the synergistic behavior of the CQDs/TiO_2_ systems rather than the intrinsic photocatalytic activity of the CQDs themselves.

**Figure 8:**
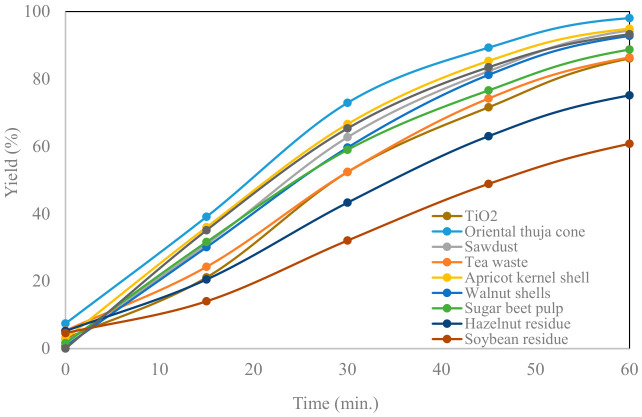
Time-dependent UV–Vis removal efficiencies of Reactive Black 5 dye by CQDs/TiO_2_ systems.

**Figure 9:**
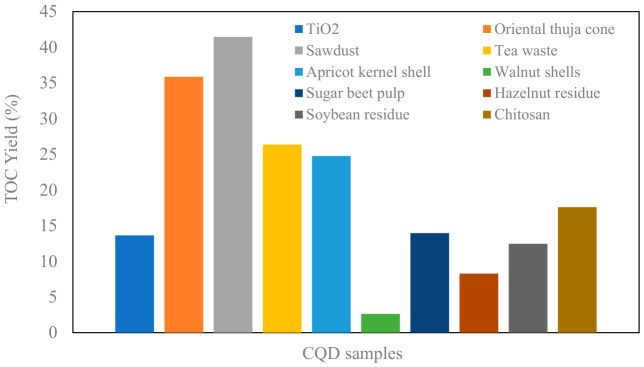
Time-dependent TOC removal efficiencies of Reactive Black 5 dye by CQDs/TiO_2_ systems.

When the photocatalytic performance of the CQDs/TiO_2_ systems presented in Figure 8 and Figure 9 is evaluated in conjunction with color removal determined by UV–Vis spectroscopy and mineralization efficiencies determined by TOC, significant differences between the two parameters are observed. The UV–Vis results indicate that the chromophore structures of the dye are rapidly broken down, and high decolorization efficiencies are achieved in most samples. In contrast, TOC removal efficiencies remain at lower levels, indicating that the systems have a limited capacity to fully mineralize the organic structure. This finding is consistent with the literature reporting that significant differences may exist between color removal and mineralization in photocatalytic degradation processes [43].

According to UV–Vis results, the highest color removal was achieved in CQDs/TiO_2_ systems based on juniper wood, apricot kernel, and chitosan (~90–98%), while the highest TOC removal performance was observed in sawdust (41.45%) and juniper wood (35.86%) samples. This clearly demonstrates that systems providing high color removal do not always exhibit the highest mineralization performance.

For example, the juniper-based system exhibited high performance in both UV and TOC aspects, demonstrating balanced photocatalytic behavior, whereas the apricot kernel and chitosan-based systems provided limited TOC removal despite high color removal.

This result indicates that the chromophore structure breaks down rapidly in these systems, but the complete oxidation of the resulting intermediates occurs more slowly. Such behavior is consistent with studies reporting that intermediate formation is common in photocatalytic processes [57].

On the other hand, a more pronounced discrepancy was observed between UV–Vis and TOC results in some samples. In particular, the soybean residue-based CQDs/TiO_2_ system, despite exhibiting one of the lowest UV–Vis removal efficiencies (~60%), demonstrated a TOC removal value of 12.46%, which is close to that of pure TiO_2_. This indicates that, although color removal is relatively limited in this system, organic compounds undergo a certain degree of oxidation. Similarly, the tea waste and apricot kernel shells exhibited moderate UV removal alongside moderate TOC removal, demonstrating a more balanced yet limited mineralization behavior.

The most notable discrepancy, however, was observed in the CQDs/TiO_2_ systems based on hazelnut residue and walnut shell. Although these samples showed moderate performance in UV–Vis removal, their TOC removal remained quite low (8.27% and 2.64%, respectively). This suggests that while the chromophore structure of the dye was effectively degraded, the resulting intermediate products accumulated within the system, thereby limiting complete mineralization.

The inclusion of pure TiO_2_ as a control system enabled direct evaluation of the contribution of CQDs to photocatalytic performance. According to the UV–Vis results, pure TiO_2_ achieved approximately 86% color removal after 60 min of irradiation. Several CQDs/TiO_2_ systems, particularly those derived from Oriental thuja cone, sawdust, apricot kernel shell, and chitosan, exhibited higher color removal efficiencies ranging from approximately 93% to 98%. A more pronounced difference was observed in the mineralization results. Pure TiO_2_ achieved a TOC removal efficiency of 13.86%, whereas the CQDs/TiO_2_ systems prepared from sawdust and Oriental thuja cone reached TOC removal efficiencies of 41.45% and 35.86%, respectively. These findings clearly demonstrate that biomass-derived CQDs can significantly enhance the photocatalytic performance of TiO_2_, not only by improving decolorization efficiency but also by promoting deeper mineralization of organic pollutants. The enhanced performance is attributed to improved charge separation, reduced electron–hole recombination, more efficient interfacial charge transfer, and enhanced generation of reactive oxidative species within the CQDs/TiO_2_ photocatalytic system.

In particular, despite the relatively high degree of surface functionalization observed in walnut shell-derived CQDs, their weak electrostatic stability and pronounced tendency toward aggregation may have reduced the effective active surface area available for photocatalytic reactions, thereby limiting mineralization performance. This observation is consistent with previous studies reporting that nanoparticle aggregation can reduce accessible active sites and hinder photocatalytic efficiency [39].

However, aggregation alone cannot fully explain the low mineralization performance. Walnut shell is characterized by a relatively high lignin content, which may promote the formation of more aromatic and graphitic carbon domains during thermal conversion. Nevertheless, graphitization degree alone does not determine photocatalytic activity. The low TOC removal efficiency observed for walnut shell-derived CQDs is likely associated with the combined effects of aggregation tendency, limited colloidal stability, surface functional group distribution, charge-transfer characteristics, and the efficiency of interfacial interactions between CQDs and TiO_2_. Therefore, the photocatalytic behavior of biomass-derived CQDs should be considered as the result of multiple interacting structural and surface-related parameters rather than a direct consequence of lignin content or graphitization degree alone.

Overall, the difference between UV–Vis removal and TOC removal is consistent with the two-step nature of photocatalytic degradation: rapid chromophore cleavage and color loss in the first step, followed by slower-progressing intermediate product oxidation and mineralization in the second step. Therefore, high color removal does not necessarily imply high photocatalytic activity on its own; TOC removal must be considered to evaluate actual performance. This two-step mechanism has been widely reported in CQDs-based photocatalytic systems [43,57].

When these findings are evaluated alongside other characterization results, it is evident that photocatalytic performance is directly related to the surface chemistry, optical properties, and colloidal behavior of CQDs.

It was determined that samples exhibiting strong π–π* and n–π* transitions in UV–Vis analyses and possessing a balanced core–surface structure demonstrated better performance, whereas in C systems showing a high tendency for aggregation or low surface charge in DLS and zeta potential results, the mineralization efficiency remained limited. This finding is consistent with studies highlighting that the surface states and dispersion properties of CQDs directly influence catalytic activity [39,45].

This finding demonstrates that effective photocatalytic performance in CQDs/TiO_2_ systems is controlled not only by light absorption but also by surface accessibility and charge carrier dynamics. This finding is consistent with studies highlighting that charge separation, surface reactions, and active site accessibility are decisive factors in photocatalytic systems [43,57].

The observed differences in photocatalytic performance can be directly attributed to the intrinsic biochemical composition of the biomass precursors. Biomass with high lignin content (e.g., walnut shell, hazelnut shell) tends to promote the formation of more graphitic and aromatic carbon areas, which enhance electron transfer and photocatalytic activity. This is consistent with the literature reporting that more developed sp^2^ carbon networks form due to the aromatic character of lignin-derived structures [39,43].

In contrast, biomass rich in cellulose and hemicellulose facilitates the formation of oxygen-containing surface functional groups, thereby increasing hydrophilicity and adsorption capacity. It is known that such surface functionalization provides advantages in terms of the adsorption of reactive species and surface reactions [38,45].

Additionally, nitrogen-rich precursors (e.g., soybean residue, chitosan) enable in situ heteroatom incorporation, creating active sites that enhance charge separation and catalytic efficiency. It has been widely reported in the literature that nitrogen doping suppresses electron–hole recombination by modifying the bandgap and enhances photocatalytic performance [45,57].

## 4. Conclusions

In this study, carbon quantum dots (CQDs) were successfully synthesized via a semi-hydrothermal method using various lignocellulosic and hemicellulosic biomass sources, and the structural, optical, colloidal, and photocatalytic properties of the resulting nanostructures were comparatively evaluated. The findings clearly demonstrated that the biomass origin is one of the most critical parameters determining the performance of CQDs.

UV–Vis analyses confirmed the presence of characteristic π–π* (250–260 nm) and n–π* (270–280 nm) transitions in all samples, indicating that CQDs formation was successfully achieved. However, the absorption intensity and spectral behavior exhibited significant differences depending on the type of biomass. In particular, the high absorbance values and broad absorption tails observed in soybean and walnut shell-based CQDs were attributed to surface defects and a high density of functional groups.

When DLS and zeta potential results were evaluated together, it was determined that the colloidal behavior of CQDs is not solely dependent on surface charge, but that steric stabilization provided by surface functional groups and biomass-derived organic components also plays a significant role. While a tendency toward aggregation was observed in most samples due to low zeta potential values, soybean residue-based CQDs exhibited strong electrostatic stability with a high negative surface charge (~−33 mV). In contrast, steric stabilization was found to be dominant in the walnut shell and apricot kernel shells.

The photocatalytic degradation experiments were performed using CQDs-modified TiO_2_ systems, where TiO_2_ served as the primary photocatalyst and biomass-derived CQDs acted as performance-enhancing components. Therefore, the observed photocatalytic behavior reflects the synergistic interaction between TiO_2_ and CQDs rather than the activity of CQDs alone.

Photocatalytic performance results revealed a significant difference between color removal (UV–Vis) and mineralization (TOC). Although high decolorization efficiencies (85–98%) were achieved in many samples, TOC removal efficiencies were found to remain at lower levels (2.6–41.4%). This is consistent with a two-step mechanism of photocatalytic degradation: rapid chromophore breakdown and a slower-progressing mineralization process.

The inconsistency between the high color removal rate and the relatively low TOC removal rate indicates that the degradation process is dominated by partial oxidation and the formation of intermediate organic species rather than complete mineralization. This suggests that CQDs enhance the photocatalytic system primarily through improved light absorption and charge transfer, but do not fully direct the mineralization pathways.

The highest mineralization efficiency was achieved in CQDs/TiO_2_ systems based on sawdust (41.45%) and thuja cone (35.86%), while very low TOC removal was observed in walnut shell (2.64%) and hazelnut residue (8.27%) samples. This situation has been attributed to high aggregation tendency, low surface charge, and limited active surface area. In contrast, it was determined that systems exhibiting a balanced core–surface structure and appropriate surface functionalization demonstrate higher photocatalytic performance.

Overall, it has been concluded that the performance of CQDs-based photocatalytic systems is governed by a multiparametric mechanism influenced by surface chemistry, optical properties, colloidal stability, and particle distribution. This study demonstrates that the selection of suitable biomass is of critical importance not only for CQDs synthesis but also for the design of high-performance photocatalytic systems for advanced environmental applications. Furthermore, the findings present a sustainable and scalable approach for converting biomass waste into high-value-added nanomaterials.

## Figures and Tables

**Table 1:** Photocatalytic Degradation Experiment Parameters.

Parameter	Test Condition
Initial dye concentration	100 ppm
Dye pH	6.84 (Neutral pH)
TiO_2_ amount	0.1 g
Volume of liquid containing carbon quantum dots	20 mL
Temperature	25 °C

**Table 2:** Proximate analysis results of biomass samples.

Sample Name	Ash (%)	Volatile Matter (%)	Fixed Carbon (%)	HHV (MJ/kg)
Oriental thuja cone	2.40 ± 0.20	73.10 ± 0.90	24.50 ± 0.70	20.01
Sawdust	1.50 ± 0.15	79.00 ± 1.00	19.50 ± 0.60	19.17
Tea waste	3.18 ± 0.25	70.60 ± 0.90	26.22 ± 0.80	20.07
Apricot kernel shell	1.10 ± 0.12	74.65 ± 0.95	24.25 ± 0.70	20.17
Walnut shells	1.30 ± 0.14	69.96 ± 0.85	28.74 ± 0.80	21.11
Sugar beet pulp	5.01 ± 0.40	67.86 ± 0.80	27.12 ± 0.80	19.99
Hazelnut residue	1.60 ± 0.15	70.06 ± 0.90	28.34 ± 0.80	20.97
Soybean residue	5.21 ± 0.45	71.16 ± 0.90	23.63 ± 0.70	19.48
Chitosan	0.60 ± 0.08	72.40 ± 0.90	27.00 ± 0.80	20.80

**Table 3:** Chemical analysis results of biomass samples.

Sample Name	Extractives (%)	Hemicellulose (%)	Cellulose (%)	Lignin (%)
Oriental thuja cone	5.2 ± 0.6	23.8 ± 1.2	42.5 ± 1.5	24.1 ± 1.3
Sawdust	4.1 ± 0.5	27.4 ± 1.3	43.2 ± 1.6	21.8 ± 1.2
Tea waste	8.4 ± 0.8	21.9 ± 1.1	33.6 ± 1.4	23.5 ± 1.2
Apricot kernel shell	6.3 ± 0.7	28.1 ± 1.4	32.8 ± 1.5	30.6 ± 1.7
Walnut shells	5.1 ± 0.6	24.7 ± 1.2	26.9 ± 1.4	39.8 ± 1.9
Sugar beet pulp	10.3 ± 1.1	27.6 ± 1.4	22.4 ± 1.2	17.9 ± 1.0
Hazelnut residue	6.1 ± 0.6	23.5 ± 1.2	33.2 ± 1.4	31.7 ± 1.6
Soybean residue	12.4 ± 1.3	21.8 ± 1.1	18.2 ± 1.0	11.6 ± 0.9
Chitosan	2.1 ± 0.3	N.A.	N.A.	N.A.

N.A.: Not applicable, since chitosan is a non-lignocellulosic biopolymer and does not contain cellulose, hemicellulose, or lignin.

**Table 4:** Elemental analysis results of biomass samples.

Sample Name	C (%)	H (%)	O (%)	N (%)	S (%)
Oriental thuja cone	49.2 ± 0.8	6.1 ± 0.2	43.7 ± 0.9	0.4 ± 0.1	BDL
Sawdust	48.6 ± 0.7	6.2 ± 0.2	44.2 ± 0.8	0.3 ± 0.1	BDL
Tea waste	46.8 ± 0.9	6.3 ± 0.2	42.9 ± 1.0	3.7 ± 0.3	BDL
Apricot kernel shell	49.8 ± 0.8	6.0 ± 0.2	43.2 ± 0.9	0.7 ± 0.1	BDL
Walnut shells	50.6 ± 0.9	6.1 ± 0.2	42.3 ± 0.9	0.6 ± 0.1	BDL
Sugar beet pulp	45.7 ± 0.9	6.5 ± 0.3	41.8 ± 1.0	3.5 ± 0.3	BDL
Hazelnut residue	49.5 ± 0.8	6.2 ± 0.2	42.7 ± 0.9	1.4 ± 0.2	BDL
Soybean residue	48.3 ± 0.9	6.8 ± 0.3	36.5 ± 1.1	7.9 ± 0.5	BDL
Chitosan	44.8 ± 0.7	6.7 ± 0.2	41.5 ± 0.9	6.5 ± 0.4	BDL

BDL: Sulfur content was below the detection limit of the CHNS analyzer for all samples.

**Table 5:** Solid product yields (wt.%) of biomass samples following thermal treatment.

Sample	Sample Name	Post-Thermal Treatment Solid Product Yield (%)
1	Oriental thuja cone	62.63 ± 0.80
2	Sawdust	67.29 ± 0.95
3	Tea waste	57.15 ± 0.70
4	Apricot kernel shell	61.91 ± 0.75
5	Walnut shells	53.89 ± 0.65
6	Sugar beet pulp	62.85 ± 0.82
7	Hazelnut residue	60.00 ± 0.78
8	Soybean residue	50.40 ± 0.60
9	Chitosan	55.54 ± 0.70

**Table 6:** Comparison of DLS Results for Biomass-Based CQDs Samples.

Sample	Source	Z-Average (nm)	PdI	Main Peak (nm)	Main Peak (%)	Secondary Peak (nm)	Secondary Peak (%)	Overall Assessment
1	Oriental thuja cone	2003	0.898	506	98.3	1853	1.7	High aggregation
2	Sawdust	1577	0.850	537	90.8	42	7.5	Multimodal distribution
3	Tea waste	3623	1.00	265	85.1	1854	14.9	Excessive aggregation
4	Apricot kernel shell	1142	0.750	3088	68.0	1747	25.4	Relatively stable
5	Walnut shells	2138	0.870	630	100	-	-	Single-peak aggregate
6	Sugar beet pulp	2281	0.880	559	100	-	-	Single-peak aggregate
7	Hazelnut residue	897	0.683	402	92.2	25	7.8	Most stable
8	Soybean residue	1033	0.651	615	85.6	1901	12.9	Good dispersion
9	Chitosan	1205	0.703	529	96.4	25	3.6	Controlled system

**Table 7:** Comparison of Zeta Potential Results for Biomass-Based CQDs.

Sample	Source	Zeta Potential (mV)	Mean ± SD (mV)	Mobility (µm·cm/V·s)	Conductivity (mS/cm)	Stability Comment
1	Oriental thuja cone	−14.7/−14.6/−15.3	−14.9 ± 0.4	−1.16	0.38	Low stability
2	Sawdust	−12.7/−14.9/−13.6	−13.7 ± 1.1	−1.08	0.25	Low stability
3	Tea waste	−6.35/−12.2/−9.97	−9.5 ± 2.9	−0.78	0.11	Very low stability
4	Apricot kernel shell	+4.39/+6.19/+6.32	+5.6 ± 1.1	+0.44	0.26	Steric effect
5	Walnut shells	−3.57/−4.53/−4.41	−4.2 ± 0.5	−0.34	0.57	Very low stability
6	Sugar beet pulp	−4.5/−4.95/−4.89	−4.8 ± 0.2	−0.38	0.85	Very low stability
7	Hazelnut residue	−7.55/−9.06/−8.08	−8.2 ± 0.8	−0.64	3.92	Steric stabilization
8	Soybean residue	−35.3/−32.2/−32.1	−33.2 ± 1.8	−2.60	5.16	High stability
9	Chitosan	−12.5/−12.2/−13.9	−12.9 ± 0.9	−1.01	0.24	Low stability

**Table 8:** UV–Vis Characteristics of CQDs Samples Produced from Biomass Sources.

Sample	Source	Aπ (250–260 nm)	An (270–280 nm)	An/Aπ	Optical Interpretation
1	Oriental thuja cone	~1.96	~1.95	~1.0	Balanced structure
2	Sawdust	~0.70	~0.53	<1	Weak core
3	Tea waste	~1.08	~0.51	<1	Medium structure
4	Apricot kernel shell	~0.38	weak	<1	Amorphous
5	Walnut shells	weak	~0.71	>1	Surface-dominant
6	Sugar beet pulp	~0.49	weak	<1	Heterogeneous
7	Hazelnut residue	~2.01	~1.75	~0.9	High activity
8	Soybean residue	~1.86	~2.3	>1	Surface-dominant
9	Chitosan	~1.96	~1.95	~1.0	Balanced

## Data Availability

The data supporting the findings of this study are available from the corresponding author upon reasonable request.

## Supplementary Materials

The following supporting information can be downloaded at: https://www.mdpi.com/article/10.3390/nano16120731/s1, Figure S1. Centrifugation process after the semi-hydrothermal treatment. Figure S2. Photodegradation reactor.
