# Supplementary material for: Biomass-Derived Carbon Quantum Dots via Semi-Hydrothermal Processing: Linking Surface Chemistry, Colloidal Stability, and Photocatalytic Mineralization Performance
Source: Nanomaterials (Basel). 2026 Jun 12;16(12):731. doi: 10.3390/nano16120731 (PMC13304770; doi:10.3390/nano16120731)
Supplement: Supplementary file 1 [file nanomaterials-16-00731-s001.zip › nanomaterials-4357308-supplementary.pdf]

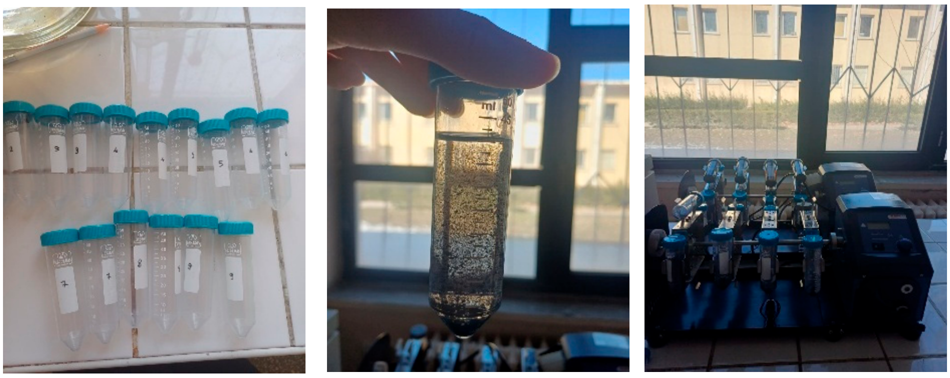

Figure S1. Centrifugation process after the semi-hydrothermal treatment

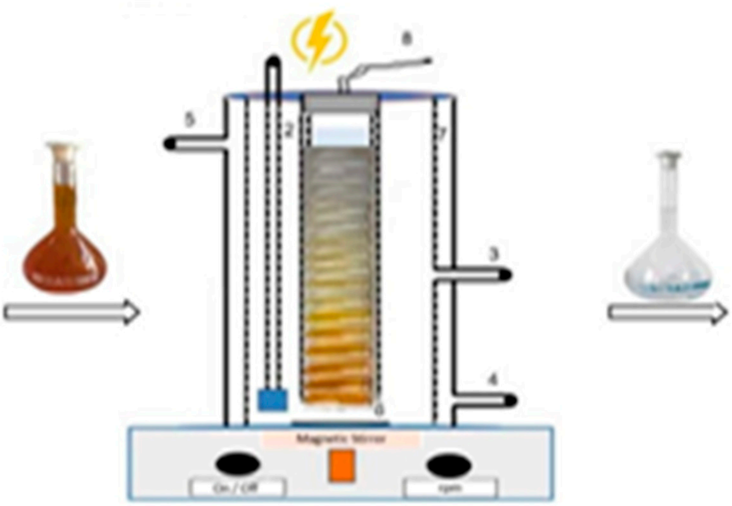

Figure S2. Photodegradation reactor
